# The Role of Sudanese Doctors in the United Kingdom in Mitigating COVID-19 Vaccine Hesitancy Among Their Diaspora Communities

**DOI:** 10.9745/GHSP-D-22-00447

**Published:** 2023-06-21

**Authors:** Ahmed Hashim, Yusri Taha

**Affiliations:** a Royal Free London NHS Foundation Trust, London, United Kingdom.; b Newcastle Upon Tyne Hospitals NHS Foundation Trust, London, United Kingdom.

## Abstract

People from ethnic minority groups are at risk of adverse sequelae of COVID-19, but their vaccine acceptance and enrollment rates remain suboptimal. Diaspora communities are unique in that they continue to engage in epistemic networks related to vaccination with dwellers in their country of origin. We describe the COVID-19 vaccine awareness campaign we conducted from January 2021 to November 2021 for Sudanese diaspora groups in the United Kingdom and the various techniques and methods used by health care professionals from the same ethnocultural background to address vaccine reluctance among this cohort. We explore the impact of our joint efforts with local authorities in Sudan to overcome the initial barriers to the national vaccination program in Sudan. The influence of the intercommunication between the diaspora and their relatives at home regarding COVID-19 vaccine misinformation is also discussed. Finally, we highlight these observations as key areas for future research to improve the global impact of vaccine deployment in pandemic situations.

## Abstract (precis)

A COVID-19 vaccine awareness campaign conducted by Sudanese doctors helped to mitigate vaccine hesitancy among Sudanese diaspora groups in the United Kingdom.

## Teaser

**Key Findings:**

- Diaspora health care professionals can assist in mitigating COVID-19 vaccine hesitancy for members of their respective diaspora communities who share similar ethnocultural characteristics and societal environments.
- The campaign’s diversification of speakers, methods, and educational platforms coupled with constant reinforcement of positive messages were critical to harnessing motivation among diaspora minorities with an engendered feeling of mistrust in the vaccine development and deployment process.

**Key Implications:**

- The unmediated interaction and crossover of false information between groups of the same ethnic and cultural backgrounds living abroad and those living in their home countries impacts global vaccine uptake and requires attention.
- The use of health care professionals from the same societal and cultural background to alleviate vaccine hesitancy among ethnic minorities should receive more support and investigation.

## BACKGROUND

People from ethnic minority groups have a well-established elevated risk of severe disease and adverse COVID-19 outcomes.^1^^–^^3^ This prompted calls, including in the United Kingdom, to prioritize vaccine rollout for these vulnerable groups.^4^ Despite consideration by the government, vaccine hesitancy prevailed among ethnic minorities, resulting in inferior enrollment rates.^5^^,^^6^ A large UK study revealed a stark disparity in vaccine uptake, with 68.3% of Black groups accepting a COVID-19 vaccine compared to 96.2% of the White population.^3^ A constellation of factors related to social, religious, epistemic, and policymaking uncertainties has likely contributed to the complexity of this issue.^5^ Vaccine skepticism and indecision were also common among ethnic minority health care workers,^7^ which added to the predicament. Health care workers often, implicitly or explicitly, fulfill the duties of community leaders and are expected to participate positively in pandemic responses.

Risk perception and demographic characteristics are important metrics that influence vaccine attitudes and behavior. While the literature generally refers to groups reluctant to receive the COVID-19 vaccine as “hesitant,” emerging evidence suggests that those who are “completely resistant” constitute a distinct group from the hesitant or indecisive cohort.^8^^,^^9^ One major study found that the 2 groups differ not only in psychosocial indicators but also in their sources and consumption of (and trust in) information about the vaccine.^9^ In fact, there is a complex continuum among the vaccine hesitant, ranging from accepting the vaccine with uncertainty to delaying acceptance to partially rejecting some of its concepts to complete refusal.^10^ Understanding this complexity is essential to appreciate how eliciting motivation to accept the vaccine through awareness campaigns, particularly among ethnic minorities, may not always translate into actual acceptance.

Understanding the complex continuum of vaccine hesitancy is essential to appreciate why awareness campaigns may not always lead to vaccine acceptance.

Evidence emerged from previous experiences indicating that immigrant populations may experience higher rates of non-COVID-19 vaccine hesitancy. For instance, the uptake rate of the measles-mumps-rubella vaccine among the Romanian diaspora in the United Kingdom was low due to cultural differences as well as convenience barriers such as difficulty in registering with general practitioners.^11^ Similarly, Somali parents in the United States were reported to exhibit less confidence in the measles-mumps-rubella vaccine.^12^ Regarding COVID-19 vaccination rates, a recent cross-sectional study revealed a lower acceptance rate among sub-Saharan African residents (14.2%) compared to their counterparts in the diaspora (25.3%).^13^ More interestingly, a report on vaccine uptake among South Sudanese communities in Canada indicated that the uptake was reasonably high and in line with the national figures, with the percentage of unvaccinated individuals estimated by community leaders to be as low as 5%–10%.^14^ Nevertheless, the report flagged concerns that vaccine acceptance by this cohort may not have been purely voluntary as some felt they were “coerced” to get vaccinated.

Since the COVID-19 vaccination campaign started, significant emphasis has been placed on strategies focused on appropriate community engagement and culturally tailored communication to help mitigate possible vaccine concerns.^15^ Community leaders and health care professionals are undoubtedly essential players in facilitating the establishment of an environment of trust within ethnic minority groups and improving their vaccine receptiveness. What has not been examined in greater depth is the role of health care practitioners in addressing these issues for diaspora groups of the same ethnic and cultural backgrounds and the impact of this on global vaccine deployment.

## COVID-19 VACCINE AWARENESS AMONG UK SUDANESE DIASPORA COMMUNITIES

### Campaign Description

The Sudanese community in the United Kingdom is considered the oldest and among the largest Sudanese diaspora groups in the Global North and includes, according to the last UK census, about 24,000 Sudanese-born residents.^16^ It is a diverse community encompassing professionals, academics, and nonskilled workers, as well as refugees and asylum seekers. In January 2021, the UK Branch of the Sudan Doctors Union (SDU-UK) launched a health awareness campaign targeting Sudanese communities in the United Kingdom. The campaign involved live, direct question-and-answer sessions and made use of various social media platforms (Box 1).^17^ It also released a series of short videos through a collaborative program between a volunteer-run, UK-based Sudanese television station and mainstream television platforms, which facilitated the distribution of educational materials to a wider audience, including viewers in Sudan (Box 1). Furthermore, a collection of illustrative posters was devised to debunk the myths surrounding COVID-19 vaccination. The posters were phrased in simple language and used the Sudanese Arabic dialect. The SDU-UK additionally combined efforts with refugee organizations to share video material on the benefits of the vaccine. Through the assistance of the Sudanese “Community Champions,” remote question-and-answer sessions were organized for local communities in the United Kingdom with high densities of refugees from Sudan and East Africa (Box 1). These sessions were particularly relevant in light of data showing that refugees in the United Kingdom have become an increasingly difficult-to-reach population for the vaccination program.^18^

BOX 1 Methods and Platforms Used to Raise Awareness and Debunk COVID-19 Vaccine Misinformation

- Live-streamed interactive sessions using the various social media platforms.
- Illustrative posters with infographics using simple language with the native dialect.
- Short, easily distributable videos addressing misconceptions/misinformation.
- Provision of technical and policymaking support to Sudan’s national COVID-19 vaccine program.
- Joint update sessions with representatives of Sudan’s Ministry of Health and the World Health Organization Sudan’s Office.

### Involvement of Sudanese Diaspora Doctors

The content of the awareness campaign was produced by expert UK-based Sudanese doctors specialized in infectious diseases, virology, and public health. Involving health care professionals from the same cultural background enables the implementation of educational programs that can conceivably diminish feelings of cultural conspiracy among vulnerable diaspora communities. In scenarios such as global vaccination campaign rollouts, trust involves 2 components: (1) the relationship between individuals and (2) the relationship between individuals and a system. The latter is linked to a long history of structural racism and medical abuses against Black populations,^19^ which is often compounded by negative personal experiences with health care systems and providers.^9^^,^^19^ Hence, among ethnic minority groups with an engendered feeling of mistrust in the authenticity of the vaccine development process, diaspora health care professionals addressing those fears with their respective ethnic communities could readily assist in enhancing vaccine uptake rates.

## CAMPAIGN APPROACHES AND CHALLENGES

### Approaches Used to Foster Vaccine Acceptance

To promote engagement with the targeted audience, the campaign occasionally adopted an approach of embedding positive vaccination messages within a broad health awareness session, beginning with the provision of general health and well-being advice before moving on to the subject of COVID-19 (Box 2). This approach avoids eliciting anxiety and resistance among listeners and captures their attention. Live-streamed interactive sessions helped break down the perceived complexity of the topic while tackling conspiracy theories surrounding COVID-19 vaccination. Conspiracy theories tend to emerge in the context of social crises, such as pandemics, when uncertainty and collective fear prevail with attempts to psychologically fill in the knowledge gap with a coherent and predictable narrative.^9^^,^^10^ Therefore, diversification of the speakers, methods, and educational platforms was vital in enabling our campaign to overcome these hurdles. Furthermore, we used constant reinforcement of positive messages to harness motivation among those disinclined to engage in the vaccination campaigns. Our program also included focused discussions on specific demographics, such as vaccinations in children and pregnant women.

BOX 2 Principles and Concepts Adopted in the COVID-19 Vaccine Educational Campaign

- Diversifying the methods of delivering the educational campaign.
- Addressing the influence of Sudanese diaspora communities on their families and friends living in their native country (and vice versa) and the crossover of misinformation between the 2 groups.
- Ensuring responsiveness, adaptability, and readiness to tackle constantly emerging misconceptions and false information.
- Embedding COVID-19 vaccination awareness messages as part of general health awareness sessions to prevent immediate rejection by the audience.
- Conducting ongoing debates and discussions among Sudanese health care professionals, particularly those who may be hesitant themselves.
- Liaising and combining efforts with community champions and refugee organizations.
- Targeting hesitancy among specific groups (children and pregnant women).

### Challenges

One of the most challenging aspects of the vaccination awareness campaign was the dynamic evolution of misinterpretations and constant emergence of data regarding the safety and efficacy of vaccines. Such developments demanded close monitoring and a swift response to address false material being promulgated in the media, as well as a high degree of adaptability and fluidity within our awareness campaign for the Sudanese community. We made an additional, peculiar observation—the intercommunication and interactions of diaspora communities with people in their native countries through readily accessible social media platforms meant that any anxieties or misinformation about the vaccines would propagate momentously and spill over from 1 group to the other, despite geographical distances. The similarity in risk perception of COVID-19 among the African diaspora in high-income countries and those living in Africa was documented in earlier studies, raising the possibility that this may also apply to COVID-19 hesitancy.^20^ Diaspora communities may retain their vaccine-related misbeliefs and concerns from before they immigrated to the host country or continue to be influenced by anti-vaccine campaigns in their native country, particularly those coming from societies engulfed with superstition and anti-science bigotry. Hence, campaign implementers should be aware of the continuous influence on the diaspora by health-related developments in their native country and vice-versa. A similar case was observed among Polish parents in the United Kingdom who appeared to have been swayed towards the rejection of the childhood influenza vaccine by a strong anti-vaccine movement in Poland.^21^ The report on COVID-19 vaccine uptake by South Sudanese diaspora in Canada also broached the notion of their engagement through online networks with families at home spreading misinformation.^14^ There is also the potential of cross-exchange of negative anti-vaccine messages between members of diaspora groups with the same background but who live in different host countries. Moreover, misinformation and rumors on social media tend to have a considerable effect on the decision to get vaccinated compared to factual information.^22^ To this end, our campaigners had to be persistent and maintain integrity in their efforts to combat negative social media influences and promote vaccine uptake.

Diaspora communities may retain their vaccine-related misbeliefs and concerns from before they immigrated or continue to be influenced by anti-vaccine campaigns in their native country.

Our doctors’ union was notably involved in supporting the COVID-19 vaccination campaign in Sudan in its initial phase and providing guidance to local health authorities since the arrival of the first batch of COVID-19 vaccines in the country.^23^ Direct collaboration with Sudan’s Ministry of Health and the World Health Organization’s Sudan Office put our health educators in an ideal situation to address the dominant misconceptions for both the Sudanese people in the United Kingdom and in Sudan. In doing so, they had to navigate through 2 social cohorts that were distinct geographically yet overlapping in terms of ethnocultural and epistemic backgrounds.

## REACH AND IMPACT OF THE CAMPAIGN

Although it was challenging to evaluate the outcomes or gauge the specific impact metrics of our vaccination campaign, pre/post questionnaires during the live-streaming sessions revealed an increase in knowledge about the vaccine and improved readiness to enroll in the vaccination program. Moreover, the social media videos were viewed by a total of over 42,000 viewers. We also observed that the reach of these sessions extended to Sudanese diaspora groups from other European countries. Locally, our conjoined endeavors with the national vaccine program in Sudan led to the successful and timely distribution of the first batch of vaccines allocated to Sudan through the World Health Organization’s COVID-19 Vaccines Global Access initiative, with 98% of the 828,000 initial doses administered before the set deadline. This enabled the country to benefit from further funding and vaccine allocations.

## CONCLUSION

Addressing COVID-19 vaccine hesitancy among diaspora and ethnic minority groups can be complex and should involve vigilant consideration of the unmediated interaction and sharing of misinformation between groups of the same ethnic and cultural backgrounds living abroad and those living in their home countries. The global impact of this synergy and interaction on vaccine uptake deserves careful attention and systematic examination. The influence of networking between similar diaspora groups in distinct geographical areas on vaccine hesitancy also merits investigation. Finally, the role of health care professionals from the same ethnocultural and societal environments in alleviating vaccine hesitancy among ethnic minorities is vital and should receive more support.

## Notes

**Peer Reviewed**

**First published online:** April 28, 2023.

## References

[1] Aldridge RW Lewer D Katikireddi SV et al. Black, Asian and Minority Ethnic groups in England are at increased risk of death from COVID-19: indirect standardisation of NHS mortality data. Wellcome Open Res. 2020;5:88. 10.12688/wellcomeopenres.15922.2. 32613083 PMC7317462

[2] Mathur R Rentsch CT Morton CE et al.; OpenSAFELY Collaborative. Ethnic differences in SARS-CoV-2 infection and COVID-19-related hospitalisation, intensive care unit admission, and death in 17 million adults in England: an observational cohort study using the OpenSAFELY platform. Lancet. 2021;397(10286):1711–1724. 10.1016/S0140-6736(21)00634-6. 33939953 PMC8087292

[3] COVID-19: review of disparities in risks and outcomes. Public Health England. June 2, 2020. Accessed April 5, 2023. https://www.gov.uk/government/publications/covid-19-review-of-disparities-in-risks-and-outcomes

[4] Osama T Razai MS Majeed A. COVID-19 vaccine allocation: addressing the United Kingdom’s colour-blind strategy. J R Soc Med. 2021;114(5):240–243. 10.1177/01410768211001581. 33689530 PMC8145446

[5] Halvorsrud K Shand J Weil LG et al. Tackling barriers to COVID-19 vaccine uptake in London: a mixed-methods evaluation [published correction appears in J Public Health (Oxf). 2022 Aug 25;44(3):725]. J Public Health (Oxf). 2022;fdac038. 10.1093/pubmed/fdac038. 35551415 PMC9383970

[6] Curtis HJ Inglesby P Morton CE et al. Recording of “COVID-19 vaccine declined” among vaccination priority groups: a cohort study on 57.9 million NHS patients’ primary care records in situ using OpenSAFELY. medRxiv. Preprint posted online August 7, 2021. 10.1101/2021.08.05.21259863

[7] Nguyen LH Joshi AD Drew DA et al. Racial and ethnic differences in COVID-19 vaccine hesitancy and uptake. medRxiv. Preprint posted online February 28, 2021. 10.1101/2021.02.25.21252402

[8] Larson HJ Broniatowski DA. Volatility of vaccine confidence. Science. 2021;371(6536):1289. 10.1126/science.abi6488. 33766861

[9] Pertwee E Simas C Larson HJ. An epidemic of uncertainty: rumors, conspiracy theories and vaccine hesitancy. Nat Med. 2022;28(3):456–459. 10.1038/s41591-022-01728-z. 35273403

[10] Kumar D Chandra R Mathur M Samdariya S Kapoor N. Vaccine hesitancy: understanding better to address better. Isr J Health Policy Res. 2016;5(1):2. 10.1186/s13584-016-0062-y. 26839681 PMC4736490

[11] Bell S Saliba V Ramsay M Mounier-Jack S. What have we learnt from measles outbreaks in 3 English cities? A qualitative exploration of factors influencing vaccination uptake in Romanian and Roma Romanian communities. BMC Public Health. 2020;20(1):381. 10.1186/s12889-020-8454-x. 32293379 PMC7092501

[12] Banerjee E Griffith J Kenyon C et al. Containing a measles outbreak in Minnesota, 2017: methods and challenges. Perspect Public Health. 2020;140(3):162–171. 10.1177/1757913919871072. 31480896

[13] Miner CA Timothy CG Percy K et al. Acceptance of COVID-19 vaccine among sub-Saharan Africans (SSA): a comparative study of residents and diasporan dwellers. BMC Public Health. 2023;23(1):191. 10.1186/s12889-023-15116-w. 36709269 PMC9884132

[14] Kur M. Attitudes of the South Sudanese diaspora towards COVID-19 vaccination in Canada. London School of Economics blog. April 18, 2022. Accessed April 11, 2023. https://blogs.lse.ac.uk/africaatlse/2022/04/18/attitudes-of-the-south-sudanese-diaspora-towards-covid-19-vaccination-in-canada

[15] Kamal A Hodson A Pearce JM. A rapid systematic review of factors influencing COVID-19 vaccination uptake in minority ethnic groups in the UK. Vaccines (Basel). 2021;9(10):1121. 10.3390/vaccines9101121. 34696228 PMC8541490

[16] Clark D. Number of Sudanese nationals resident in the United Kingdom from 2016 to 2021. Statista. Accessed April 5, 2023. https://www.statista.com/statistics/1253327/sudanese-population-in-united-kingdom/

[17] Our COVID-19 Campaign. Sudan Doctors Union UK Branch. Accessed April 5, 2023. https://sdu.org.uk/?page_id=1990

[18] Deal A Hayward SE Huda M et al; ESCMID Study Group for Infections in Travellers and Migrants (ESGITM). Strategies and action points to ensure equitable uptake of COVID-19 vaccinations: a national qualitative interview study to explore the views of undocumented migrants, asylum seekers, and refugees. J Migr Health. 2021;4:100050. 10.1016/j.jmh.2021.100050. 34075367 PMC8154190

[19] Wise J. Pfizer accused of testing new drug without ethical approval. BMJ. 2001;322(7280):194. 10.1136/bmj.322.7280.194. 11159610 PMC1119465

[20] Abu EK Oloruntoba R Osuagwu UL et al. Risk perception of COVID-19 among sub-Sahara Africans: a web-based comparative survey of local and diaspora residents. BMC Public Health. 2021;21(1):1562. 10.1186/s12889-021-11600-3. 34404377 PMC8370831

[21] Tankwanchi AS Bowman B Garrison M Larson H Wiysonge CS. Vaccine hesitancy in migrant communities: a rapid review of latest evidence. Curr Opin Immunol. 2021;71:62–68. 10.1016/j.coi.2021.05.009. 34118728

[22] Loomba S de Figueiredo A Piatek SJ de Graaf K Larson HJ. Measuring the impact of COVID-19 vaccine misinformation on vaccination intent in the UK and USA. Nat Hum Behav. 2021;5(3):337–348. 10.1038/s41562-021-01056-1. 33547453

[23] Sudan receives first delivery of COVID-19 vaccines with over 800 000 doses. World Health Organization Regional Office for the Eastern Mediterranean. March 3, 2021. Accessed April 11, 2023. http://www.emro.who.int/media/news/sudan-receives-first-delivery-of-covid-19-vaccines-with-over-800-000-doses.html

